# Relationships Between Aerobic Performance, Hemoglobin Levels, and Training Load During Small-Sided Games: A Study in Professional Soccer Players

**DOI:** 10.3389/fphys.2021.649870

**Published:** 2021-02-16

**Authors:** Saeid Younesi, Alireza Rabbani, Filipe Manuel Clemente, Rui Silva, Hugo Sarmento, António José Figueiredo

**Affiliations:** ^1^University of Coimbra, Research Unit for Sport and Physical Activity, Faculty of Sport Sciences and Physical Education, Coimbra, Portugal; ^2^Department of Exercise Physiology, Faculty of Sport Sciences, University of Isfahan, Isfahan, Iran; ^3^Escola Superior Desporto e Lazer, Instituto Politécnico de Viana do Castelo, Rua Escola Industrial e Comercial de Nun’Álvares, Viana do Castelo, Portugal; ^4^Instituto de Telecomunicações, Delegação da Covilhã, Covilhã, Portugal

**Keywords:** football (soccer), athletic performance, aerobic capacity, drill-based games, SSGs, motor skills

## Abstract

The purposes of this study were (1) to analyze between-session variations of external and internal load measures during small-sided games (SSGs) and (2) to test the relationships between the maximum speed reached (V_IFT_) during the last stage of the 30-15 Intermittent Fitness Test, hemoglobin levels, and training load measures during SSG intervals among professional soccer players. Sixteen professional soccer players (mean ± SD; age 27.2 ± 3.4 years, height 174.2 ± 3.6 cm, body mass 69.1 ± 6.4 kg, and body fat 10.4 ± 4.1%) participated in this study. Hemoglobin and aerobic performance were first tested, and then a 3-week SSG program was applied using a 3 vs. 3 format. During those 3 weeks, internal and external load of entire sessions were also monitored for all training sessions. Trivial-to-small, standardized differences were observed between sessions for external and internal measures during SSGs. Total distance (TD) and mechanical work (MW) were the only variables that indicated small changes. Large-to-very-large relationships were found between V_IFT_ and external loads: TD (*r* range: 0.69; 0.87), high-intensity running (HIR; *r* range: 0.66; 0.75), and MW (*r* range: 0.56; 0.68). Moderate-to-large negative relationships were found between hemoglobin levels and internal loads: Edwards’ TRIMP (*r* range: −0.36; −0.63), %HR_max_ (*r* range: −0.50; −0.61), and red zone (*r* range: −0.50; −0.61). V_IFT_ had unclear relationships with overall internal loads, while hemoglobin levels presented unclear relationships with overall external loads. In conclusion, no meaningful changes were found between sessions considering the format of play used. Additionally, the detected relationships indicate that V_IFT_ and hemoglobin levels are good indicators of the performance capacity and physiological profile of players during SSGs. Also, the use of SSGs protocols as a monitoring complement of the 30-15_IFT_ is suggested.

## Introduction

The practical operationalization of soccer training in recent years has placed great emphasis on the inclusion of small-sided games (SSGs), which encompass the psychological, physical, technical, and tactical aspects of the game (Clemente et al., 2012). For coaches, the main objective of this training method is to mimic all those aspects of soccer that are required during competitions but on a smaller scale (Clemente et al., 2020; Clemente and Sarmento, 2020). Given SSGs’ widespread use among coaches and practitioners, researchers have examined the external demands and internal responses of different SSG drills (Clemente et al., 2014; Arslan et al., 2017). Because of the SSG’s beneficial and ecological use, there is a tendency to leave out the traditional running-based conditional exercises during a soccer training session (Moran et al., 2019). However, some research suggests that SSGs themselves may not be enough to promote the same patterns of the required physical demands during a soccer match, mainly due to the reduced frequency of high-intensity distance-based metrics of this training approach (Hammami et al., 2017; Lacome et al., 2017). Knowing the physical demands during SSGs is, for that reason, an important way to ensure a proper stimulus on the players.

The assessment and monitoring of a soccer player’s current physiological and performance status assume an imperative role for better decision-making during the training process (Drew and Finch, 2016). Although there are several field testing methods used for assessing a player’s performance capacity, these methods tend to be time-consuming, and coaches tend to reject these types of assessments given their maximal or sub-maximal nature (Turner et al., 2011). Researchers, however, have analyzed the relationships between traditional aerobic fitness tests with external and internal training load measures during SSGs, as well as the capacity of SSGs to assess the intermittent aerobic fitness of soccer players (Owen et al., 2020). In an interesting study (Owen et al., 2020) using a 5v5 format, it was found a moderate-to-very large and large-to-very-large associations between external and internal measures, as well as with aerobic performance (in the Yo-Yo Intermittent Recovery test [YYIR], respectively. In that same study, the authors also found low coefficient of variation (CV%) values in external and internal measures, suggesting the potential use of the assessed 5v5 format protocol as an intermittent aerobic fitness assessment tool for soccer players. Furthermore, it seems that YYIR’s strongest relationships are with total distance and high-intensity running metrics during soccer matches (Aquino et al., 2020). Considering SSGs, moderate-to-large correlations were observed when examining the relationships between external loads during 6v6 drills and YYIR performance (Stevens et al., 2016). Also, moderate-to-very large negative relationships were reported between heart rate measures, such as time spent above 80% HR_max_ (red zone), %HR_max_, during SSGs and YYIR performance (Stevens et al., 2016; Owen et al., 2020).

Not only physical performance can be determinant for optimizing performance in SSGs, but also biological conditions. Examples as hematological parameters could be crucial for predicting optimum physical performance since hemoglobin and red blood cells play a fundamental role in transporting oxygen (Schumacher et al., 2002). In this regard, it has been widely demonstrated that endurance training produces adaptations at the blood level. Such fact is characterized by an increase in blood volume explained by an expansion in the volume of plasma and an increase in the number of red blood cells (Sawka et al., 2000). The results of that study demonstrate that players residing at moderate altitudes have greater concentrations of hemoglobin (16.2 ± 0.2 g·dl^−1^) than players not living at sea-level locations (14.4 ± 0.7 g·dl^−1^). The mean VO2max value of the players residing at a moderate altitude (54.1 ml·kg^−1^·min^−1^) was also significantly greater than that of the players residing at low altitudes (49 ml·kg^−1^·min^−1^; Sawka et al., 2000). One review demonstrated that a higher hemoglobin concentration is related to an improvement in physical performance of between 5 and 10% (Wilber, 2002). The results of this review imply that soccer players residing at low altitudes should travel to the higher regions for a few days prior to the start of the pre-season period. This is because erythropoiesis begins the first day an individual is at a higher altitude. In approximately 4–7 days, this translates into an increase in hemoglobin concentration (Klausen et al., 1991). Recently, it has been suggested that assessments of the total mass of hemoglobin can be used as a predictor of VO2max in athletes, as it can help to identify talents in endurance sports (Eastwood et al., 2009).

However, to the best of our knowledge, there is no study addressing the relationships between the 30-15_IFT_, hemoglobin concentrations, and different external and internal load measures during SSG drills among professional players. The potential relationships between those variables can assist coaches and practitioners in using SSGs as a more ecological and time-efficient monitoring tool of player’s physiological profile and performance capacity and might also serve as a complement to traditional running-based assessments. Additionally, several studies have analyzed between-session variations in the external and internal load measures of SSGs and suggested the use of standardized differences and small worthwhile change (SWC) to analyze the degree of changes/differences in performance between athletes instead of using percentages of changes (Buchheit and Rabbani, 2014; Clemente et al., 2019b; Younesi et al., 2021). Thus, it is important to understand how the load during SSGs may vary from session to session. Therefore, the purposes of the present study were (1) to analyze between-session variations of external and internal load measures during SSGs and (2) to analyze the relationships between V_IFT_, hemoglobin levels, and training loads measures during SSG intervals among professional soccer players. It is hypothesized that selected internal and external load measures will present no meaningful changes between sessions and that a possible relationship will be observed between training load in SSGs and the physical and hematological characteristics of the players.

## Materials And Methods

### Study Design and Settings

This study followed an observational analytic cohort design. Players were assessed during the 1st week of data collection for hemoglobin levels and aerobic performance. After that, 3 weeks of training sessions were monitored, in which repeated-measures design was tested for specific SSGs (3v3), employed with the same conditions (same players and teams, with the same number of resting days in between). The data collection occurred in 2018/2019, in the first 4 weeks of the pre-season. However, training time (between 17:00 and 20:00) and environmental conditions (ambient temperature and relative humidity, ranging between 25 and 32°C and 54 and 76%, respectively) varied greatly over the data-collection phase. Data related to external load measures were obtained using global positioning systems during all SSG sessions, while internal load was monitored using heart rate monitors. All players involved in the study were professionals and were familiar with SSGs prior to the experimental period.

### Participants

Sixteen professional soccer players (mean ± SD; age 27.2 ± 3.4 years, height 174.2 ± 3.6 cm, body mass 69.1 ± 6.4 kg, and body fat 10.4 ± 4.1%, 3.1 ± 1.5 years in the club), all members of a professional club competing in the 2018–2019 season of Qatar Star League (Qatar first division), participated in this study. The inclusion criteria were (i) participation in all the moments of assessment and games; (ii) absence of injuries, physical constraints, or illnesses exhibited during sessions occurred in the period and two weeks prior to the data collection; and (iii) absence of signals of fatigue on assessment days. Players were assigned to different teams (of three elements), and comparisons between teams revealed no meaningful changes in the main outcome (aerobic performance). The baseline fitness levels of players revealed an average V_IFT_ of 17.9 ± 1.2 km/h. All players were informed of the experimental procedures and related risks and gave informed consent before commencing the study. The study protocol was approved by the University Research Ethics Committee. The study followed the ethical standards of the Declaration of Helsinki.

### Small-Sided Games

The 3v3+Gk SSG format was used in the present study. This format was repeated over three trials of three *m*, with two sets of three minutes performed in each session. The conditions involved touch limitations (a maximum of three consecutive touches permitted to each player) in accordance with a previous study suggesting ball limitation as a way to increase the intensity (Dellal et al., 2011). Three working intervals were implemented for the 3v3 + GK format. Two minutes were allotted for recovery between intervals. Pitch dimensions were 20 × 27 m for 3v3, as the playing areas were standardized (~90 m^2^ per player, excluding the goalkeeper).

The coaching staff, which included one of the authors (a strength and conditioning coach), were directed to ensure the consistency of their supervision during all SSG sessions. Balls were kept near the SSG pitch so that coaches could restart the game immediately if a ball left the playing area. For each format, the teams were balanced based on their members’ physical and technical abilities (as determined by the coach); this reduced the possibility of any strength or weakness bias.

### Data Measurement and Quantitative Variables

#### Internal Load Measurement

Heart rate data were recorded during all SSG sessions using Bluetooth HR sensors (Polar H10, Polar-Electro, Kempele, Finland; recorded in 5-s intervals) that were synchronized to a validated portable 10-Hz VX Sport 350 GPS units (VX Sport, Wellington, New Zealand). Each unit was used for the same player across the study to reduce inter-unit variability. HR measures, including HR_max_, Edwards’TRIMP, and time spent in the red zone (>80% of individual HR_max_), were analyzed following each session. The HR_max_ of each individual was extracted from a maximal field-based test (i.e., the 30-15 Intermittent Fitness Test) conducted during the pre-season. To standardize Edwards’ TRIMP and red zone measures so that fair comparisons, the corresponding data were divided by minutes played (Edwards’TRIMP.min^−1^ and Red zone.min^−1^).

#### External Load Measurement

External load measures were recorded during all sessions using portable 10-Hz VX Sport GPS units (VX Sport, Wellington, New Zealand), which are valid and reliable according to Buchheit et al. (2014). External load measures included total distance (TD), high-intensity running (HIR, distance > 14.4 km.h^−1^), and mechanical work (MW), for which the numbers of acceleration and deceleration efforts above and below 2.2 m.s^2^ thresholds were calculated the sum. The thresholds used for acceleration/deceleration efforts (2.2 m.s^2^) were selected based on practical experiences using the VX GPS system by the coaching staff. All external load measures were standardized by being divided by minutes played (e.g., TD.min^−1^) prior to the analysis so that they could be compared across different SSG formats.

#### Hemoglobin

A blood sample (15 ml) was collected from each participant between 8:00 and 10:00 a.m. The samples were collected with an overnight fast and after a minimum of 12 h of rest (without exercise). Blood samples were centrifuged at 2500 rpm for 10 min, and the serum of each sample was stored. Hemoglobin (g/dl) was determined using flow cytometry.

#### The 30-15 Intermittent Fitness Test

This test consisted of 30-s shuttles interspersed by 15-s periods of passive recovery. The velocity was set at 8 km.h^−1^ for the first run, and speed was increased by 0.5 km/h for each subsequent run. Players had to run back and forth between two lines positioned 40 m apart. Running pace was set by an automatic beeper to control the running speed when players entered a 3-m zone placed in the middle and at both extremities of the field. During the recovery period, players walked toward the closest line, either at the middle or one of the ends of the running area, depending on where they stopped in the last run. Players were told to complete as many stages as possible, and the test was ended when the players could not maintain the required running speed or could not reach the 3-m zone before the beep three consecutive times. The final velocity registered in the last stage determined the player’s V_IFT_ score (Buchheit et al., 2009; Grgic et al., 2020).

## Statistical Analysis

Data are presented as mean and standard deviation (SD) or 90% of confidence limits (CL) where specified. Differences between sessions in terms of training load measures were analyzed using standardized differences or effect size (ES; Cohen, 2013). Qualitative thresholds for interpreting the ES were as follows: <0.2 = *trivial*, <0.60 = *small*, <1.2 = *moderate*, <2.0 = *large*, and ≥2.0 = *very large* (Hopkins et al., 2009). A magnitude-based inference approach using the smallest worthwhile difference or change (SWC, 0.2 × between-subject SD) was used to analyze the likelihood that the true changes were clear. Pearson’s correlation coefficients were used to determine the relationships between V_IFT_ and hemoglobin levels with internal and external load measures during SSG. Qualitative thresholds for correlations were categorized as follows: <0.1 = *trivial*, <0.3 = *small*, <0.5 = *moderate*, <0.7 = *large*, <0.9 = *very large*, and ≤1.0 = *near perfect* (Hopkins et al., 2009). If the 90% confidence intervals of the Pearson’s correlation coefficients and standardized difference overlapped by small positive and negative values (±0.1 and ±0.2 for *r* and ES, respectively), the relationship was deemed *unclear*; otherwise, the obtained magnitude was deemed to be the observed magnitude (Hopkins et al., 2009). The statistical analysis was performed using a dedicated Excel spreadsheet built by Hopkins (2015).

## Results

The mean ± SD values of players’ V_IFT_ and hemoglobin were 18.0 ± 1.2 km.h^−1^ and 14.5 ±0.7 g.dl^−1^, respectively. The mean ± SD of external and internal load measures during different sessions/sets of SSGs are represented in Table 1.

**Table 1:** Mean ± SD of external and internal training load measures during small-sided games.

	Session 1		Session 2		Session 3	
Measure	Set 1	Set 2	Set 1	Set 2	Set 1	Set 2
TD (m)	355.6 ± 50.0	361.1 ± 44.2	352.7 ± 45.2	366.1 ± 44.4	375.3 ± 49.1	384.7 ± 63.2
HIR (m)	53.3 ± 25.9	54.1 ± 24.9	52.9 ± 24.5	54.9 ± 23.6	55.2 ± 27.0	56.9 ± 24.1
MW (n)	14.3 ± 4.9	15.0 ± 4.3	15.2 ± 4.6	15.1 ± 3.5	17.4 ± 6.8	17.1 ± 6.2
TRIMP (AU)	10.9 ± 1.0	10.8 ± 1.0	10.9 ± 1.0	10.9 ± 1.0	10.8 ± 1.0	11.2 ± 0.9
Red zone (min)	2.1 ± 0.5	2.0 ± 0.5	2.0 ± 0.5	2.0 ± 0.4	2.0 ± 0.4	2.1 ± 0.4
% of HR_max_	80.8 ± 4.8	81.3 ± 4.7	81.1 ± 4.9	81.5 ± 4.6	81.6 ± 4.8	81.8 ± 4.9

TD: total distance; HIR: high-intensity running (>14.4 km.h^−1^); MW: mechanical work (the number of accelerations and decelerations >2.2 m.s^2^); TRIMP: Edwards’ Training Impulse; AU: arbitrary units; Red zone: time spent in red zone (>80% of maximal heart rate); HR_max_: maximal heart rate; SD: standard deviation.

### Between-Session Differences in External and Internal Load Measures

**Figure 1:**
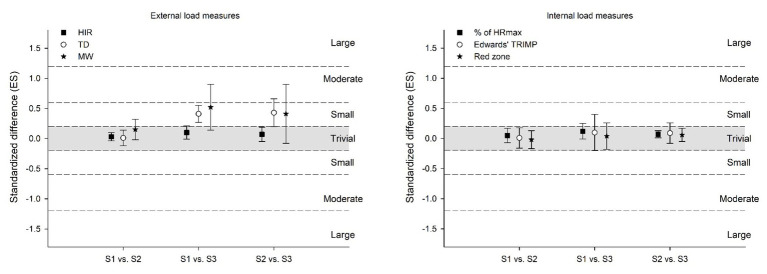
Standardized differences between sessions in external and internal load measures during small-sided games. TD: total distance; HIR: high-intensity running (>14.4 km.h^−1^); MW: mechanical work (the number of accelerations and decelerations >2.2 m.s^2^); Edwards’ TRIMP: Edwards’ Training Impulse; Red zone: time spent >80% of maximal heart rate; HR_max_: maximal heart rate; ES: effect size.

*Trivial*-to-*small* standardized differences were observed in TD (ES, 01; 0.43) and MW (ES, 0.15; 0.52) between sessions (Figure 1). *Trivial* (ES, 03; 0.10) changes were observed in HIR between sessions (Figure 1). *Trivial* changes were also observed in all internal load measures, including Edwards’ TRIMP (ES, 0.01; 0.10), time spent in the red zone (ES, −0.02; 0.06), and average HR as % of HR_max_ (ES, 0.05; 0.12) between sessions (Figure 1).

### Relationship Between V_IFT_ and Training Load Measures During SSGs

**Figure 2:**
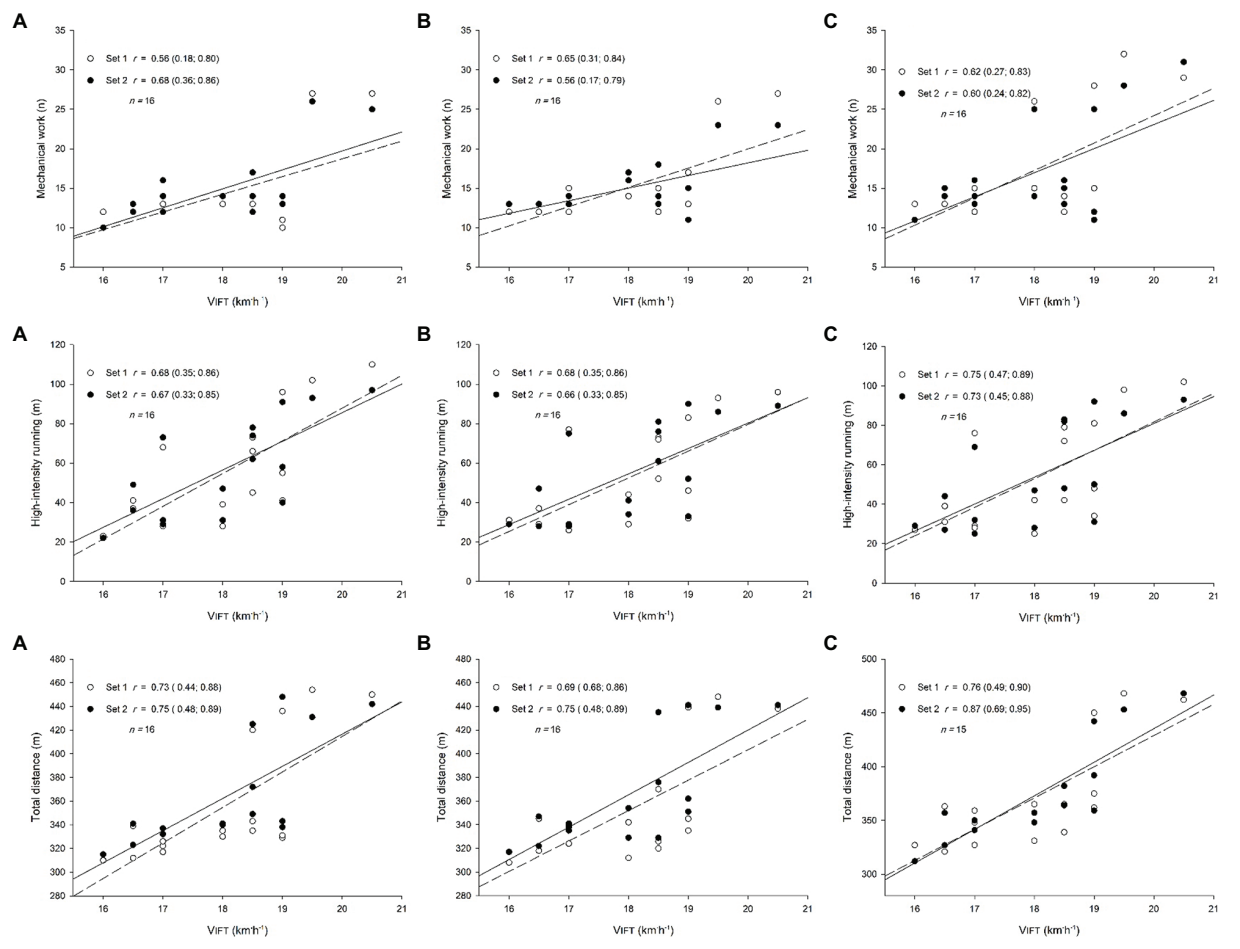
Relationships (correlation coefficient, *r*, 90% confidence limits) between the final speeds reached at the end of the 30–15 Intermittent Fitness Test (V_IFT_) and external load measures during small-sided game intervals. **(A)**: Session 1, **(B)**: Session 2, **(C)**: Session 3. High-intensity running: distance covered above14.4 km.h^−1^; Mechanical work: the number of accelerations and decelerations > 2.2 m.s^2^.

*Large*-to-*very-large* positive correlation coefficients were observed between V_IFT_ with TD (*r* range: 0.69; 0.87) and HIR (*r* range: 0.66; 0.75) in different sessions (Figure 2). V_IFT_ also showed *large* positive relationships (*r* range: 0.56; 0.68) with MW in different sessions (Figure 2). However, *small* negative to *unclear* positive associations were observed between V_IFT_ and all internal load measures (*r* range: −0.25; 0.26).

### Relationship Between Hemoglobin Levels and Training Load Measures During SSGs

**Figure 3:**
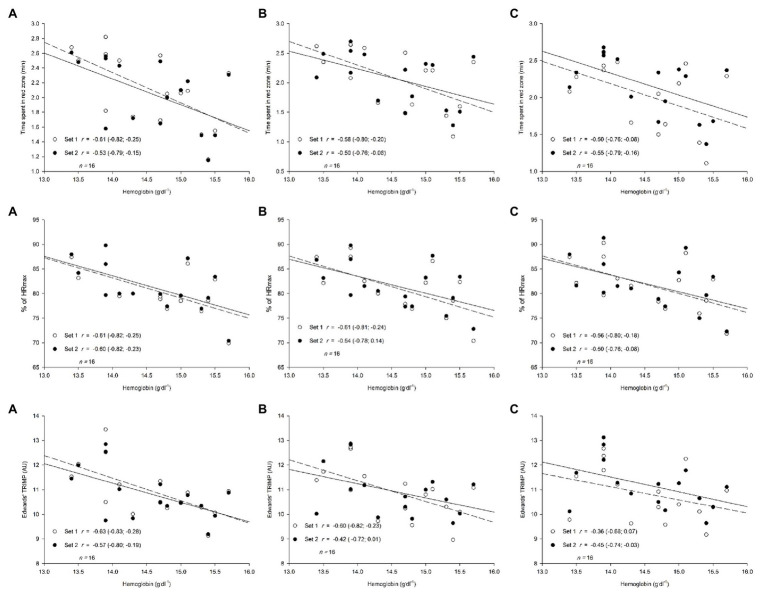
Relationships (correlation coefficient, *r*, 90% confidence limits) between players’ hemoglobin levels (g.dl^−1^) and internal load measures during small-sided game intervals. **(A)**: Session 1, **(B)**: Session 2, **(C)**: Session 3. Edwards’ TRIMP: Edwards’ Training Impulse; AU: arbitrary units; Time spent in red zone; time spent >80% of maximal heart rate; HR_max_: maximal heart rate.

Inversed *moderate*-to-*large* associations were observed between hemoglobin levels and Edwards’ TRIMP (*r* range: −0.36; −0.63; Figure 3). *Large* inverse relationships were observed between hemoglobin levels and average HR (as a % of HR_max_), as well as time spent in the red zone (*r* range: −0.50; −0.61) in different sessions (Figure 3). The relationships between hemoglobin levels and external load measures were all *unclear*, as they included negative to positive *trivial*-to-*moderate* correlations (*r* range: −0.30; 0.29). The only *clear* associations observed between hemoglobin levels and external load measures were the *moderate* correlations observed when considering HIR in the second sets of the first and second sessions (*r* range: 0.38; 0.40).

## Discussion

This study was designed to analyze between-session standardized differences of external and internal load measures and the relationships between V_IFT_ and hemoglobin levels with training loads during SSGs intervals among professional soccer players. The main findings were that the external measures had trivial-to-small standardized differences, while all internal measures revealed trivial standardized differences between sessions. V_IFT_ showed large-to-very-large positive relationships with TD, HIR, and MW, while hemoglobin levels showed moderate-to-large negative relationships with TRIMP, time spent in red zone, and %HR_max_ during different sessions. However, V_IFT_ presented unclear relationships with all internal loads, while hemoglobin levels presented unclear relationships with overall external load.

Between-session standardized differences of SSGs were more pronounced only for TD and MW when comparing session 1 and 2 to 3, representing a small degree of change. Meanwhile, HIR presented trivial changes between all sessions. Using different approaches, other study have analyzed between-sets variations of SSGs and reported trivial-to-small standardized differences from the first set to the second set for TD when considering SSGs shorter and longer regimens (Clemente et al., 2019a). Also, the same pattern was reported for TD, HIR, and MW measures when considering different SSG formats (3v3, 4v4, and 6v6) with and without goalkeepers and with and without touch limitations (Younesi et al., 2021). Some explanations for the variations in external load demands are the variability of individual and collective dynamics related to tactical behavior (Clemente et al., 2020). However, in smaller formats, the individual participation is higher, thus possible variability may decrease. Interesting internal load measures remained consistent across sessions, which are of paramount importance for chronic adaptations to occur (Buchheit and Laursen, 2013). Similarly, Clemente et al. (2019a) found that the average HR measure presented trivial-to-small changes in both shorter and longer SSG regimens between three different sets, revealing HR consistency. However, that study was conducted on amateur soccer players, and the authors analyzed only HR average between-sets changes. The present study analyzed other HR measures and between-session changes, which makes the comparison with our results somewhat complex. From this, neither the external loads nor the internal loads seem to vary between-sessions during this type of SSGs formats.

The large-to-very-large relationships between V_IFT_ and TD measures revealed in the present study are in line with other study that demonstrated a strong association between Yo-Yo IR scores and total distance covered and high-intensity running speeds during match play, suggesting a strong validity (Aquino et al., 2020). In fact, in a study conducted on 23 elite soccer players with the aim to analyze the relationships between a 5v5 SSG and the YYIR1 running performance, it was found that TD during the SSGs was very strongly associated with YYIR1 performance (*r* = 0.88; Owen et al., 2020). In our study, the final 30-15_IFT_ score (V_IFT_) revealed similar associations with TD (*r* range: 0.69; 0.87) during SSG drills. Also, in the study of Stevens et al. (2016), the largest association was observed between YYIR2 and TD (*r* = 0.59) during 6v6 SSGs when considering only the elite population that was analyzed in their study. Considering the HIR measure, moderate-to-large (*r* = 0.45; 0.56) relationships were previously reported between YYIR2, YYIR1, and the time spent in HIR during a match (Rebelo et al., 2014). The present study revealed large-to-very-large associations between V_IFT_ and HIR (*r* = 0.66; 0.75). In the same way, Stevens et al. (2016) reported large associations between HIR and YYIR2. This is of great interest, as it was previously demonstrated that HIR is one of the best variables to discriminate between won and lost matches (Aquino et al., 2018). In fact, despite differences between smaller formats (3vs.3) and larger formats as reported in previous studies (Owen et al., 2012), it seems that even in this case (3vs.3) physical fitness plays an important role since the magnitude of correlations.

Furthermore, the MW measures had large relationships with V_IFT_ in the present study. These results contrast with a previous study that reported only a moderate relationship without statistical significance between acceleration measure and YYIR2 during 6v6 SSGs. However, there is a possible explanation for this difference. In fact, shorter formats of play are characterized by more changes of direction and accelerations/decelerations, thus being in line with something that 30-15_IFT_ is also sensitive to (i.e., detecting the change-of-direction ability; Haydar et al., 2011). Our results also corroborate other work (Owen et al., 2020) that reported moderate-to-large associations between decelerations and accelerations and YYIR1. However, in that study, those metrics showed low R-squared values (R^2^ = 27 and 2% for accelerations and decelerations, respectively). This could mean that MW measures contain a higher factor of unexplainable variability – in fact, previous studies have reported a higher coefficient of variation for those metrics within SSGs (Ade et al., 2014; Aquino et al., 2019; Younesi et al., 2021). However, these comparisons must be carefully analyzed because our study considered MW measure, which encompasses both accelerations and decelerations, while other reported studies analyzed those metrics separately. Given the large-to-very-large associations between V_IFT_ and the analyzed external measures considered during the SSGs in our study, it can be suggested that the V_IFT_ is a good indicator of a player’s performance during SSGs. Also, the examined SSGs may be used as a soccer-specific monitoring tool for the assessment of performance capacity.

Despite that, V_IFT_ showed unclear associations with all internal measures. In fact, this is in contrast with other studies that examined the relationships between YYIR (levels 1 and 2) and HR measures during SSGs, which reported large-to-very-large inversed correlations (Stevens et al., 2016; Owen et al., 2020). Similar to our results are those from the study of Aquino et al. (2019), who reported unclear associations between average HR during SSGs and average HR during official matches. Notwithstanding the use of V_IFT_ as a useful performance indicator, its capacity to assess internal loads remains unclear, assuming that internal loads do not necessarily indicate the running physical performance.

On the other hand, hemoglobin levels had moderate-to-large negative relationships with all internal loads during SSGs. However, unclear relationships with all external loads were found. To the best of our knowledge, there no study has reported possible relationships between hemoglobin levels and internal and external loads during SSGs, with only one study (Saidi et al., 2019) having examined (among other variables) the associations between hemoglobin levels and YYIR1 performance. According to our results, hemoglobin levels seem to decrease as the intensity of an SSG drill increases. In fact, increases in hemoglobin levels are related to a greater aerobic performance due to the better oxygen transport capacity (Otto et al., 2013). Several studies reported declines in hemoglobin levels in professional soccer players after periods of high-intensity activity (Silva et al., 2008; Bekris et al., 2015). Given that, monitoring hemoglobin levels can be useful for assessing SSGs intensity. Also, it may be a good indicator of players’ fatigue, as their physical performance seems to decrease after congested weeks, resulting in a decrease of hematological parameters, including hemoglobin levels (Saidi et al., 2019). Furthermore, in the same study, which was conducted on 18 elite soccer players and analyzed the relationships between hemoglobin levels and YYIR1 performance, reported only small, insignificant correlations without statistical significance (*r* = 0.21; *p* = 0.12). These results are similar to our results with regard to the unclear associations between hemoglobin levels and the external loads during SSGs (Saidi et al., 2019).

The present study is not without its limitations. For one, the small sample size makes it difficult to make any generalizations based on the results. However, the sample comprised professional players, who make up an interesting population for ensuring the quality of the results presented here. Other study limitation can be related with variation in environmental conditions occurred during the period of intervention. A final study limitation can be related to the temporal displacement between the analysis of aerobic performance and the last session of SSGs.

Regardless, as far as we know, the present study is the first to analyze the relationships between V_IFT_, hemoglobin levels, and training load measures among professional soccer players during SSG intervals. Therefore, since SSGs are highly specific and frequently used throughout the season, they could be a useful fitness indicator because (i) more players can be evaluated at the same time, (ii) SSGs involve technical and tactical demands, and (iii) players are performing soccer training during the test. As practical implications, this study suggests that the internal load and external load of SSGs (considering the outcomes used) are stable across the sessions, while training load is associated with the fitness and physiological status of the players. Based on that, coaches should consider adjusting the stimulus or drills to specific groups of players.

## Conclusion

The present study revealed low between-sessions standardized differences for the overall internal and external measures of SSGs, with only TD and MW showing small changes. Thus, it is suggested that tactical behaviors might interfere with those metrics during SSGs. V_IFT_ is a good indicator of performance capacity during SSGs, given the large-to-very-large relationships between and external measures. Also, hemoglobin levels seem to decrease at higher intensity drills, revealing associations with internal loads. Despite that, V_IFT_ and hemoglobin levels revealed unclear relationships with internal and external measures, respectively. For those reasons, it is suggested that practitioners consider both internal and external loads. It is also suggested that they use SSGs to monitor soccer players’ physiological and performance capacity to complement periodic assessment protocols such as the 30-15_IFT_.

## Data Availability Statement

The raw data supporting the conclusions of this article will be made available by the authors, without undue reservation.

## Ethics Statement

The studies involving human participants were reviewed and approved by University of Coimbra. The patients/participants provided their written informed consent to participate in this study.

## Author Contributions

SY, AR, FC, and AF lead the project, collected the data, treated the data, wrote the statistical report, and revised the original manuscript. RS and HS wrote and revised the original manuscript. All authors contributed to the article and approved the submitted version.

### Conflict of Interest

The authors declare that the research was conducted in the absence of any commercial or financial relationships that could be construed as a potential conflict of interest.
